# Antioxidative Effects of Ascorbic Acid and Astaxanthin on ARPE-19 Cells in an Oxidative Stress Model

**DOI:** 10.3390/antiox9090833

**Published:** 2020-09-06

**Authors:** Sanghyeon Oh, Young Joo Kim, Eun Kyoung Lee, Sung Wook Park, Hyeong Gon Yu

**Affiliations:** 1 Interdisciplinary Program in Stem Cell Biology, Graduate School of Medicine, Seoul National University, Seoul 03080, Korea; OhSaHy@hotmail.com; 2 Department of Ophthalmology, Seoul National University Hospital Biomedical Research Institute, Seoul 03080, Korea; yjkim612@daum.net; 3 Department of Ophthalmology, Seoul National University College of Medicine, Seoul National University Hospital, Seoul 03080, Korea; righthanded8282@gmail.com (E.K.L.); academypark@gmail.com (S.W.P.)

**Keywords:** antioxidant, ascorbic acid, astaxanthin, oxidative stress, diabetic retinopathy, retinal pigment epithelium, retinal disease

## Abstract

Oxidative stress has been implicated as critical pathogenic factors contributing to the etiology of diabetic retinopathy and other retinal diseases. This study investigated antioxidative effect of ascorbic acid and astaxanthin on ARPE-19 cells within an oxidative stress model induced by common biological sources of reactive oxygen species (ROS). Hydrogen peroxide (H_2_O_2_) at concentrations of 0.1–0.8 mM and 20–100 mJ/cm^2^ of ultraviolet B (UVB) were treated to ARPE-19 cells. Cell viability and intracellular ROS level changes were measured. With the sublethal and lethal dose of each inducers, 0–750 μM of ascorbic acid and 0–40 μM of astaxanthin were treated to examine antioxidative effect on the model. Ascorbic acid at concentrations of 500 and 750 μM increased the cell viability not only in the UVB model but also in the H_2_O_2_ model, but 20 and 40 μM of astaxanthin only did so in the UVB model. The combination of ascorbic acid and astaxanthin showed better antioxidative effect compared to each drug alone, suggesting a synergistic effect.

## 1. Introduction

Diabetic retinopathy (DR) is a microvascular consequence of diabetes mellitus and remains the leading cause of blindness among the working-age population [1]. DR is defined as the progressive, irreversible deterioration of retinal microvasculature as a result of chronic hyperglycemia [2]. Studies have found the relationship between oxidative stress and DR that oxidative stress plays a role in pathogenesis of DR and DR can increase the reactive oxygen species (ROS) level. Hyperglycemia is thought to be one of the main causes of the disease and higher level of oxidative stress can accelerate the process by blocking the downstream flow of glycolysis [3,4]. DR also increases oxidative stress because high glucose level and retinal vascularization by diabetic induction elevate arginase activity which later increases oxidative stress [5].

As the relationship between ROS and various retinal pathogenesis have been studied, defense mechanisms against ROS have been also studied [6,7,8,9,10,11]. Organisms have defense mechanisms against oxygen metabolites and the mechanism includes removal of free radicals by enzymes, proteins, and pro-oxidant metal reactions, and reduction of free radicals by antioxidants (vitamin C, vitamin E, glutathione) [6]. The cellular antioxidant response element is essentially important for the amelioration of oxidative stress. It responds to hyperglycemia and can be used to evaluate the complications of diabetes. In a previous study, Busik et al. [12] suggested that diabetes-related endothelial injury in the retina may be due to glucose-induced cytokine release by other retinal cells, such as retinal pigment epithelium (RPE) and Müller cells, and not a direct effect of high glucose. Therefore, it is important to investigate the oxidative stress as well as effects of antioxidants on RPE cells in order to determine the pathogenesis regarding oxidative stress in DR.

Studies have found that with aging, endogenous antioxidants level [13] and antioxidant enzyme activity along with its gene expression and protein level decrease [14]. This alteration in the antioxidative defense system worsens the imbalance between ROS production and its removal. As a consequence, oxidatively damaged macromolecules including lipids, deoxyribonucleic acid (DNA), and proteins accumulate accelerating the aging process with oxidative-stress-induced aging [15].

For this reason, it becomes more important to maintain the antioxidant defense system and one way is to supplement antioxidants from an outer source. Supplements actively studied for their antioxidative effect are ascorbic acid (vitamin C), glutathione, alpha-tocopherol (vitamin E), and other carotenoids (i.e., astaxanthin, lutein, β-carotene) [16,17,18]. One frequently used way to evaluate their antioxidant activity is by studying their reactivity with free radicals and metal ions (DPPH, ABTS, FRAP, CUPRAC, ORAC, HORAC, TRAP) [19,20,21,22]. However, giving them enough credence for their antioxidant capacity assumption is often controversial since one same antioxidant can have a different relative capacity to other antioxidants when measured with different methods [23,24,25,26,27,28].

For this reason, it is necessary to study potential antioxidants’ capacities and properties based on a solid oxidative stress model. A solid oxidative stress model portrays the biological environment well so that a more accurate assumption is possible, and the result is reproducible. Hydrogen peroxide (H_2_O_2_) [29,30,31] and ultraviolet B (UVB) irradiation [32,33,34] have been studied to establish an oxidative stress model within cells. H_2_O_2_ represents endogenous ROS production and UVB represents an outer source of oxidative stress to retinal cells. In this study, both a H_2_O_2_-induced oxidative stress model and UVB-induced oxidative stress model will be used to evaluate the antioxidative potential of ascorbic acid and astaxanthin on ARPE-19 cells.

## 2. Materials and Methods

### 2.1. ARPE-19 Cell Culture

ARPE-19 cells (american type culture collection, Manassas, VA, USA) were cultured and maintained as a monolayer in 1:1 mixture of Dulbecco’s modified eagle’s medium and nutrient mixture F-12 (DMEM/F-12) (Invitrogen, Gibco, Carlsbad, CA, USA) supplemented with 10% fetal bovine serum (FBS) (Invitrogen) and 1% penicillin-streptomycin (Invitrogen). Cells were incubated at 37 °C in a humidified 5% CO_2_ incubator in the complete medium with a 2–3-times-a-week change until they reached 80% confluency. Cells used for this study were in a passage between 25 and 30.

### 2.2. Hydrogen Peroxide Exposure Procedure

Cells were seeded in a 96-well plate with a density of 2.5 × 10^4^ cells/well and allowed to attach to the bottom of the well and to become confluent overnight. The next day, the medium was changed to a serum-free medium and cells were maintained in it up to 7 days until the day of the procedure. 30% (*w*/*w*) H_2_O_2_ in H_2_O-containing stabilizer (Sigma Aldrich, St. Louis, MO, USA) was used to make medium with intended H_2_O_2_ concentration. H_2_O_2_ solution was diluted fresh each time. For the exposure, the used medium of the cells was changed to serum-free DMEM/F-12 without phenol red (Invitrogen) with the desired concentration of H_2_O_2_. The viability was checked by MTT assay after 24 h of exposure to H_2_O_2_.

### 2.3. Ultraviolet B Irradiation Procedure

Cells were seeded in a 96-well plate with a density of 2.5 × 10^4^ cells/well and allowed to attach to the bottom of the well and to become confluent overnight. The next day, the medium was changed to serum-free medium and cells were maintained in it up to 7 days until the day of the procedure. At UVB irradiation, the medium was changed to DMEM/F-12 without phenol red without serum. As a UVB source, Sankyo Denki lamps (G15T8E, Tokyo, Japan) was used. Its irradiation intensity was 0.2 mW/cm^2^ when measured 20 cm below the lamp where the plates were put. The intensity was measured with a UVB meter (UVX Digital Radiometer, UVP, Upland, CA, USA). Cells were irradiated with intended doses of UVB and for the control group and differential dose of UVB irradiation, remaining wells in the same plate were thoroughly masked.

### 2.4. DPPH Scavenging Assay

Total antioxidative capacities of ascorbic acid and astaxanthin were estimated using DPPH (2,2-diphenyl-1-picrylhydrazyl) ROS scavenging assay. DPPH solution was made by dissolving DPPH in methanol to 0.16 mM. Ascorbic acid and astaxanthin were dissolved to various concentrations in dimethyl sulfoxide (DMSO) (Sigma Aldrich). Ascorbic acid (20 μL) or astaxanthin (20 μL) solution was mixed with 100 μL DPPH solution for 30 min with vigorous shaking at room temperature. After the reaction absorbance at 517 nm was measured and the relative amount of scavenged DPPH was calculated using the following equation.

(1) $$\mathrm{Scavenged}\;\mathrm{DPPH}\;\mathrm{fraction}\;\left(\%\right)=\frac{Ab_{\mathrm{Control}}-Ab_{\mathrm{AO}}}{Ab_{\mathrm{Control}}}\times 100$$

$Ab_{\mathrm{Control}}$ is the absorbance of the groups with only DPPH and $Ab_{\mathrm{AO}}$ is the absorbance of the groups of the mixture of DPPH and various concentrations of antioxidants.

### 2.5. Antioxidant Treatment

Cells were treated with either ascorbic acid (Sigma Aldrich) or astaxanthin (Sigma Aldrich) in DMEM/F-12 without phenol red to study their antioxidative effect on ARPE-19 cells. Ascorbic-acid-containing medium was made from ascorbic acid stock (0.5 M in PBS) and astaxanthin-containing medium was made from astaxanthin stock (1 mg/mL in DMSO). Cells were pretreated with ascorbic acid or astaxanthin for 6 h and then they were irradiated by UVB or exposed to H_2_O_2_. For UVB irradiation group, after pretreatment, used medium was changed to the fresh medium containing the same concentrations of compounds and followed the UVB irradiation (20 mJ/cm^2^ or 100 mJ/cm^2^) procedure. For the H_2_O_2_ exposure group, after pretreatment, the used medium was changed to the fresh medium containing the same concentrations of the compounds with a sublethal or lethal dose of H_2_O_2_ (0.2 mM or 0.4 mM). 

### 2.6. MTT Assay

3-(4,5-Dimethyl-2-thiazolyl)-2,5-diphenyl-2H-tetrazolium bromide (MTT) (Sigma Aldrich) was used to determine cell viability. MTT is enzymatically turned into purple formazan crystals by mitochondrial respiration activity. The procedure was done following the manufacturer’s instructions. Briefly, after antioxidants, UVB, or H_2_O_2_ treatment to the cells, the medium was removed and MTT (0.5 mg/mL) was added diluted in serum-free medium. After 3 h of incubation at 37 °C in a humidified 5% CO_2_ incubator, MTT-containing medium was carefully aspirated from the well and DMSO was added to each well to solubilize formazan crystals. Absorbance at 570 nm was measured using a microplate reader (EPOCH 2, BioTek Instruments Inc. Winoosky, VT, USA) with a reference wavelength of 630 nm. Cells untreated or treated with the only vehicle were set to be 100% cell viability for the normalization of the absorbance and experiments had more than three replicates for each condition.

### 2.7. Crystal Violet Assay

The relative number of cells attached to the bottom of the well was measured by crystal violet uptake assay. The procedure was done as previously described [35]. Briefly, after UVB, or H_2_O_2_ treatment to the cells, the medium was removed, and cells were fixed with 4% paraformaldehyde in 4 °C. After they were washed 3 times and 0.1% crystal violet (Sigma Aldrich) in 10% ethanol was added to each well for 5 min. After washing 3 times, the remaining stain was dissolved in 10% acetic acid and absorbance at 540 nm was measured.

### 2.8. DCFH-DA Intracellular ROS Level Assay

Intracellular ROS level was measured by 2′,7′-dichlorodihydrofluorescein diacetate (DCFH-DA) assay. DCFH-DA is cell-permeable and is not fluorescent which enters cells to be de-esterified to 2′,7′-dichlorodihydrofluorescein (DCFH), and become impermeable to the cell membrane. It then reacts with ROS to be highly fluorescent 2′,7′-dichlorofluorescein (DCF). Before UVB irradiation or H_2_O_2_ exposure, cells were cultured with 10 μM DCFH-DA (Sigma Aldrich) in DMEM/F-12 without phenol red for 30 min at 37 °C in a humidified 5% CO_2_ incubator. After incubation, they were washed 2 times in phosphate-buffered saline (PBS) and antioxidant treatment, UVB irradiation or H_2_O_2_ exposure was done following measurement of fluorescence of DCF at excitation and emission wavelength of 495 nm and 529 nm, respectively, with a microplate reader (Synergy Mix, BioTek Instruments Inc. Winoosky, VT, USA). Cells untreated or treated with the only vehicle were set to be 100% intracellular ROS level for the normalization of the fluorescence intensity and experiments had more than three replicates for each condition. 

### 2.9. Statistical Analysis

The results were expressed as mean values and standard deviation (SD). Statistical analyses were performed using the Kruskal-Wallis test for comparison between several groups and the Mann-Whitney *U* test for comparison between 2 subgroups to assess the effects of drug treatment, with *p* < 0.05. The analyses were done using IBM SPSS Statistics for Windows, Version 26.0 (IBM Corp., Armonk, New York, NY, USA).

## 3. Results

### 3.1. Effect of H_2_O_2_ on the Viability of ARPE-19 Cells and Intracellular ROS Level

**Figure 1:**
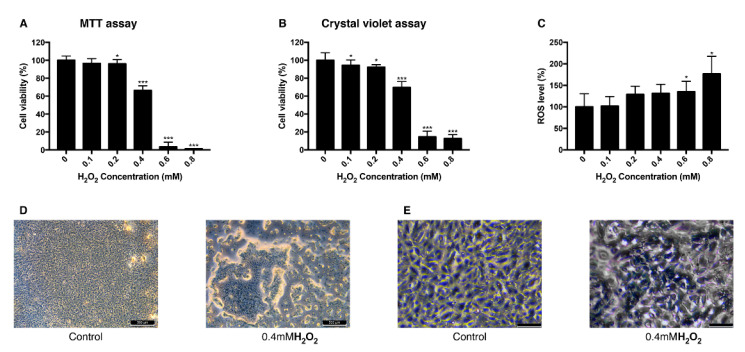
Change of viability and intracellular ROS level in ARPE-19 cells after exposure to H_2_O_2_. The response of ARPE-19 cells to 0–0.8 mM H_2_O_2_ exposure for MTT assay (**A**), and crystal violet assay (**B**) to determine cell viability. For intracellular ROS level, DCFH-DA was treated for 30 min after the H_2_O_2_ exposure. Exposure to H_2_O_2_ reduced the cell viability (**A**,**B**) and increased the intracellular ROS level (**C**). The cell morphology was observed with bright field microscopy (Scale bar 500 μm) (**D**) and with higher magnification (scale bar 100 μm) (**E**). Asterisks indicate a significant reduction in cell viability or increment in ROS level compared with untreated cells (* *p* < 0.05, ** *p* < 0.01, *** *p* < 0.001). MTT, 3-(4,5-dimethylthiazol-2-yl)-2,5-diphenyltetrazolium bromide; ROS, reactive oxygen species; DCFH-DA, 2′,7′-dichlorodihydrofluorescein diacetate.

To establish the H_2_O_2_-induced oxidative stress model in ARPE-19 cells, different concentrations of H_2_O_2_ were treated to the cells and their viability and intracellular ROS level were measured. Viability measured with MTT assay decreased as the concentration of treated H_2_O_2_ increased. When cells were treated with 0.4 mM H_2_O_2_, they showed the viability of 66% and the viability change was the greatest between 0.2 mM and 0.6 mM (Figure 1A). Crystal violet assay resulted in a similar aspect of viability change as MTT assay with 69% of viability at 0.4 mM (Figure 1B). Intracellular ROS level increased dependently to the concentration of H_2_O_2_ (Figure 1C). The mean value of the ROS level measured in 0.8 mM H_2_O_2_ increased to 176% compared to the control group. This trend of decreased cell viability after the H_2_O_2_ exposure was confirmed in bright field imaging (Figure 1D,E). As cell viability changed rapidly at 0.4 mM H_2_O_2_, 0.4 mM was set to be a lethal dose of H_2_O_2_ and 0.2 mM was set to be sublethal dose.

### 3.2. Effect of UVB Irradiation on the Viability of ARPE-19 Cells and Intracellular ROS

**Figure 2:**
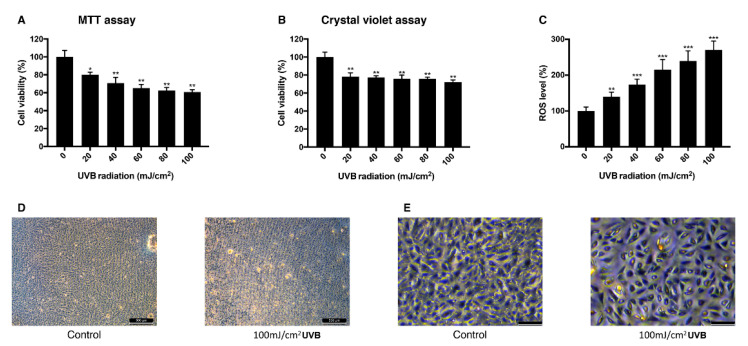
Change of viability and intracellular ROS level in ARPE-19 cells after UVB irradiation. The response of ARPE-19 cells 24 h after 0–100 mJ/cm^2^ UVB irradiation with MTT assay (**A**), and crystal violet assay (**B**) to determine cell viability. For intracellular ROS level, DCFH-DA was treated for 30 min after the UVB irradiation. Irradiation by UVB reduced the cell viability (**A**,**B**) and increased the intracellular ROS level (**C**). The cell morphology was observed with bright field microscopy (scale bar 500 μm) (**D**) and with higher magnification (scale bar 100 μm) (**E**). Asterisks indicate a significant reduction in cell viability or increment in ROS level compared with untreated cells (* *p* < 0.05, ** *p* < 0.01, *** *p* < 0.001). UVB, ultraviolet B; MTT, 3-(4,5-dimethylthiazol-2-yl)-2,5-diphenyltetrazolium bromide; ROS, reactive oxygen species; DCFH-DA, 2′,7′-dichlorodihydrofluorescein diacetate.

To establish the UVB-induced oxidative stress model in ARPE-19 cells, different doses of UVB were exposed to the cells and their viability and intracellular ROS level were measured. Viability measured with MTT assay decreased as the dose of UVB irradiation increased. When cells were exposed to 20 mJ/cm^2^ UVB, they showed the viability of 80% and with 100 mJ/cm^2^ UVB, the viability was 60% (Figure 2A). In a crystal violet assay with the same range of UVB dose, the viability dropped to 78% at 20 mJ/cm^2^ UVB and to 72% at 100 mJ/cm^2^ UVB (Figure 2B). Intracellular ROS level increased dependently to the UVB dose (Figure 2C). The mean value of ROS level measured at 20 mJ/cm^2^ UVB increased to 140% and 270% at 100 mJ/cm^2^ UVB compared to the control group. Morphological change of the cells was observed in bright field imaging. Cells became rounder and holes in the monolayer were observed as UVB dose increased (Figure 2D,E). The sublethal dose of UVB was set to be 20 mJ/cm^2^, where the cells show 80% of viability without significant morphological change and 100 mJ/cm^2^ where the cells show 60% of viability with morphological change was set to be the lethal dose of UVB. 

### 3.3. Antioxidative Effect of Ascorbic Acid and Astaxanthin by Scavenging DPPH

**Figure 3:**
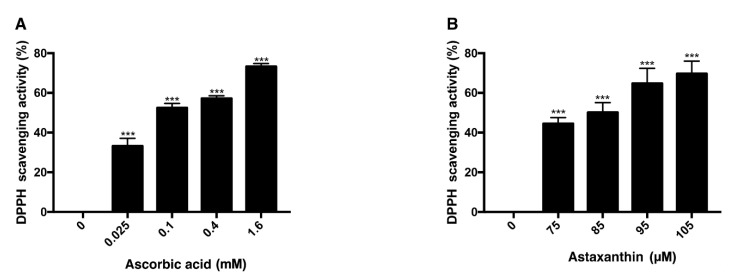
DPPH scavenging activity of ascorbic acid and astaxanthin. The antioxidative capacities of ascorbic acid and astaxanthin were determined by their capabilities to scavenge DPPH. Ascorbic acid (0.025–1.6 mM) was reacted with DPPH (**A**), and astaxanthin (75–105 μM) was reacted with DPPH (**B**). The compounds were diluted in DMSO. Both compounds scavenged DPPH in dose-dependent way in 30 min of reaction time. Asterisks indicate a significant increment in DPPH scavenging activity compared with controls (*** *p* < 0.001). DPPH, 2,2-diphenyl-1-picrylhydrazyl; DMSO, dimethyl sulfoxide.

DPPH scavenging assay was performed with ascorbic acid and astaxanthin. Ascorbic acid, at concentrations of 0.025 mM, 0.1 mM, 0.4 mM, and 1.6 mM, dissolved in DMSO was mixed with DPPH solution and each concentration scavenged 33%, 52%, 57%, 73% of DPPH, respectively, after 30 min of reaction (Figure 3A). When 75 μM, 85 μM, 95 μM, and 105 μM astaxanthin dissolved in DMSO were reacted with DPPH solution for 30 min, 44%, 50%, 64%, and 69% of DPPH were scavenged, respectively (Figure 3B). Both ascorbic acid and astaxanthin showed antioxidative effect.

### 3.4. Antioxidative Effect of Ascorbic Acid on ARPE-19 Cells Under H_2_O_2_-Induced Oxidative Stress

**Figure 4:**
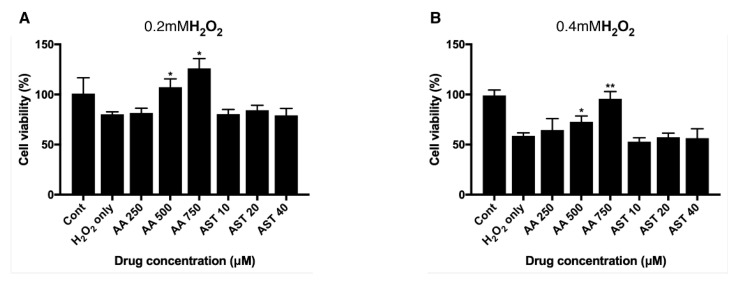
Effect of ascorbic acid and astaxanthin on H_2_O_2_-induced oxidative stress model of ARPE-19. The effect of various concentration of ascorbic acid or astaxanthin (pretreated for 6 h and co-treated with H_2_O_2_ for 24 h) on the response of ARPE-19 cells to sublethal dose of 0.2 mM (**A**) or lethal dose of 0.4 mM H_2_O_2_ (**B**). The cell viability was determined by MTT assay. Treatment of ascorbic acid (500–750 μM) significantly increased ARPE-19 cell viability following 0.2 mM H_2_O_2_ exposure. However, astaxanthin (10–40 μM) did not significantly affect the cell viability (**A**). Ascorbic acid (500–750 μM) also significantly increased the cell viability under 0.4 mM H_2_O_2_ but astaxanthin (10–40 μM) did not have significant effect on the viability (**B**). Asterisks indicate a significant increment in cell viability compared with cells treated with H_2_O_2_ only (* *p* < 0.05, ** *p* < 0.01). AA, ascorbic acid; AST, astaxanthin; MTT, 3-(4,5-dimethylthiazol-2-yl)-2,5-diphenyltetrazolium bromide.

ARPE-19 cells were pretreated with various concentrations of ascorbic acid or astaxanthin for 6 h and then they were treated together with H_2_O_2_ and the same concentrations of antioxidants for another 24 h. Viability was assessed after 3 h of MTT treatment. When groups treated together with ascorbic acid and H_2_O_2_ they showed increased viability compared to controls. Cells treated only with 0.2 mM H_2_O_2_ showed the viability of 80% and groups treated together with ascorbic acid showed 81%, 107%, and 126% of viability, respectively, for 250 μM, 500 μM, and 750 μM of the drug concentration. On the other hand, astaxanthin did not show any significant effect on the viability of ARPE-19 with H_2_O_2_-induced oxidative stress (Figure 4A). For 0.4 mM H_2_O_2_ treatment, cells treated only with H_2_O_2_ showed 58% of viability, while 250 μM, 500 μM, and 750μM of ascorbic acid increased the viability to 64%, 72%, and 95%, respectively. On the other hand, astaxanthin did not show any significant effect on the viability of ARPE-19 with H_2_O_2_-induced oxidative stress (Figure 4B). 

### 3.5. Antioxidative Effect of Ascorbic Acid and Astaxanthin on ARPE-19 Cells Under UVB-induced Oxidative Stress

**Figure 5:**
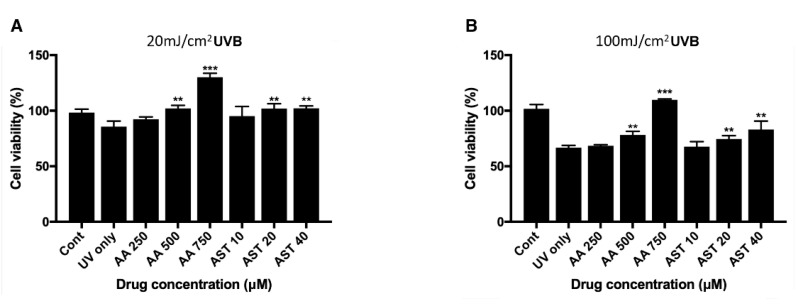
Effect of ascorbic acid and astaxanthin on UVB-induced oxidative stress model of ARPE-19. The effect of various concentration of ascorbic acid and astaxanthin (pretreated for 6 h and additional 24 h after UVB irradiation) on the response of ARPE-19 cells to sublethal dose of 20 mJ/cm^2^ (**A**) or lethal dose of 100 mJ/cm^2^ UVB (**B**). The cell viability was determined by MTT assay 24 h after the irradiation. Treatment of ascorbic acid (500–750 μM) and astaxanthin (20–40 μM) significantly increased ARPE-19 cell viability following 20 mJ/cm^2^ UVB irradiation (**A**). Ascorbic acid (500–750 μM) and astaxanthin (20–40 μM) also significantly increased the cell viability after 100 mJ/cm^2^ UVB irradiation (**B**). Asterisks indicate a significant increment in cell viability compared with cells treated with UVB only (* *p* < 0.05, ** *p* < 0.01, *** *p* < 0.001). UVB, ultraviolet B; AA, ascorbic acid; AST, astaxanthin; MTT, 3-(4,5-dimethylthiazol-2-yl)-2,5-diphenyltetrazolium bromide.

ARPE-19 cells were pretreated with various concentrations of ascorbic acid or astaxanthin for 6 h and then they were irradiated with UVB. Viability 24 h after the irradiation was assessed with MTT assay. When cells were pretreated with ascorbic acid and then UVB irradiated with it, the cell viability increased compared to the UVB irradiation-only group. Cells irradiated only with 20 mJ/cm^2^ UVB showed the viability of 85% and groups treated together with ascorbic acid showed 92%, 102%, and 130% of viability, respectively, for 250 μM, 500 μM, and 750 μM of the drug concentration. Astaxanthin treated cells also showed increased viability compared to the cells irradiated only with UVB. The 10 μM, 20 μM, and 40 μM astaxanthin groups showed 95%, 101%, and 102%, respectively, after 20 mJ/cm^2^ UVB irradiation (Figure 5A). For 100 mJ/cm^2^ UVB irradiation, the cells irradiated only with UVB showed 66% of viability while 250 μM, 500 μM, and 750 μM of ascorbic acid increased the viability to 68%, 78%, and 109%, respectively. Astaxanthin-treated cells also showed increased viability compared to the cells irradiated only with UVB. The 10 μM, 20 μM, and 40 μM astaxanthin groups showed 67%, 74%, and 83% after 100 mJ/cm^2^ UVB irradiation (Figure 5B). 

### 3.6. Effect of Ascorbic Acid on the Intracellular ROS Level of ARPE-19

**Figure 6:**
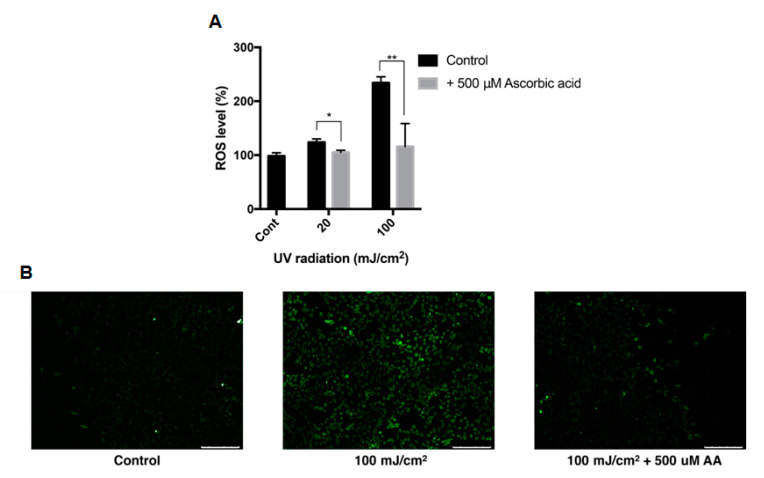
Intracellular ROS level of ARPE-19 after UVB treatment with ascorbic acid. The effects of ascorbic acid on the intracellular ROS level of ARPE-19 under UVB-induced oxidative stress were examined by DCFH-DA assay. Ascorbic acid at 500 μM significantly reduced the ROS level after UVB irradiation (20–100 mJ/cm^2^) compared to groups with UVB irradiation only (**A**). The green fluorescence of the reacted DCFH-DA which indicates the ROS level, was observed with fluorescence microscopy (scale bar 250 μm) (**B**). Asterisks indicate a significant reduction in ROS level compared with control cells only with UVB exposure without ascorbic acid treatment (* *p* < 0.05, ** *p* < 0.01). ROS, reactive oxygen species; UVB, ultraviolet B; DCFH-DA, 2′,7′-dichlorodihydrofluorescein diacetate; AA, ascorbic acid.

The effect of ascorbic acid on the intracellular ROS level of ARPE-19 cells was studied with DCFH-DA assay. The intracellular ROS level was measured after cells were treated with UVB with or without 500 μM ascorbic acid. UVB of 20 mJ/cm^2^ and 100 mJ/cm^2^ increased the intracellular ROS level to 123% and 234%, respectively, and 500 μM ascorbic acid treatment reduced the ROS level to 105% and 115% (Figure 6A). This trend between groups were confirmed with fluorescence microscopy (Figure 6B).

### 3.7. Antioxidative Effect of Astaxanthin and Ascorbic Acid by Reducing Intracellular ROS in ARPE-19 Cells

**Figure 7:**
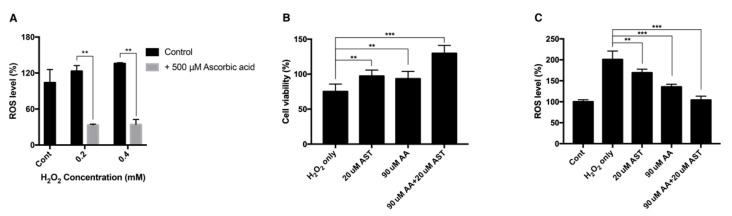
Intracellular ROS level and cell viability of ARPE-19 after H_2_O_2_ exposure with ascorbic acid and the mixture of ascorbic acid and astaxanthin. Ascorbic acid at 500 μM significantly reduced the intracellular ROS level under sublethal and lethal dose of H_2_O_2_ (0.2–0.4 mM) compared to the control group without ascorbic acid treatment (**A**). The effect of 20 μM astaxanthin, 90 μM ascorbic acid, and the mixture of the two compounds on the cell viability of ARPE-19 under H_2_O_2_-induced oxidative stress was examined by MTT assay. Cell viability was significantly increased when the cells were pretreated with 20 μM astaxanthin, 90 μM ascorbic acid, and the mixture of the two compounds for 6 h and with 0.2 mM H_2_O_2_ for 24 h, compared to H_2_O_2_ only (**B**). ROS level was significantly decreased when the cells were pretreated with 20 μM astaxanthin, 90 μM ascorbic acid, and the mixture of the two compounds for 6 h and with 0.2 mM H_2_O_2_ for 24 h, compared to H_2_O_2_ only. Asterisks indicate a significant difference between increment in cell viability and reduction in intracellular ROS level compared to control cells only with H_2_O_2_ exposure without antioxidant treatment (**C**). (** *p* < 0.01, *** *p* < 0.001). AST, astaxanthin; AA, ascorbic acid; ROS, reactive oxygen species; MTT, 3-(4,5-dimethylthiazol-2-yl)-2,5-diphenyltetrazolium bromide.

H_2_O_2_ of 0.2 mM and 0.4 mM increased the intracellular ROS level to 123% and 135% compared to the nontreated group, while ascorbic-acid-treated group showed reduced ROS level of 33% and 34%, respectively (Figure 7A). 

ARPE-19 cells were pretreated with either 20 μM astaxanthin, 90 μM ascorbic acid, or a mixture of 20 μM astaxanthin and 90 μM ascorbic acid. When cells were exposed to 0.2 mM H_2_O_2_ for 24 h, the viability decreased to 75%. The 20-μM-astaxanthin- and 90-μM-ascorbic-acid-treatment could increase the viability to 97% and 93%, respectively. The mixture of 20 μM astaxanthin and 90 μM ascorbic acid increased the viability to 129% (Figure 7B). Each drug could also decrease the intracellular ROS level. When cells were treated with 0.2 mM H_2_O_2_ for 24 h, the intracellular ROS level increased to 200%. The 20-μM-astaxanthin- and 90-μM-ascorbic-acid-treatment reduced the ROS level to 169%, and 135%, respectively. The mixture of 20 μM astaxanthin and 90 μM ascorbic acid decreased the ROS level to 104% (Figure 7C).

## 4. Discussion

In this study, antioxidative properties of ascorbic acid and astaxanthin were evaluated based on H_2_O_2_-induced and UVB-induced oxidative stress models within ARPE-19 cells. Studies have found that H_2_O_2_ and UVB have different effects on cells regarding oxidative stress. First, even directly adding H_2_O_2_ in the cell culture medium results in a short-term exposure because its concentration decreases rapidly in the presence of the cells. H_2_O_2_ can penetrate the cell easily, but it is also reduced rapidly by the antioxidative mechanism [29]. On the other hand, UVB has a lingering effect on the cells by directly damaging DNA, causing gene mutation, and modifying gene expression, and enzyme activity along with increasing ROS level [36]. UVB-induced damage is mediated by two different pathways. sne is by ROS generated immediately after the irradiation and the other is by reactive nitrogen species in the later time point [37]. As a result, even with a single and momentary exposure to UVB, the viability of the exposed cells decreases in the course of time [36].

Based on the precedent research, viability change of ARPE-19 cells was evaluated after 24 h for a H_2_O_2_ model and a UVB model [38]. H_2_O_2_ (0–0.8 mM) was exposed to ARPE-19 cells and their viability was dose-dependently reduced and intracellular ROS level was increased. UVB also reduced the cell viability and increased the intracellular ROS level but the H_2_O_2_ seemed to decrease the viability exponentially. One explanation can be that because H_2_O_2_ not only produces ROS, but it also affects junctional integrity of the RPE cell [39], weakening the cell adhesion to the bottom of the well—the cell viability assay result may have been affected. This can also explain the lower cell viability at 0.4 mM of H_2_O_2_ than 40 mJ/cm^2^ UVB even though cells with H_2_O_2_-induced oxidative stress have a lower ROS level.

Within the condition of sublethal and lethal doses of H_2_O_2_ and UVB, antioxidative potencies of ascorbic acid and astaxanthin were evaluated. Although their antioxidative properties had been studied and have moved on to clinical level with patients with retinal diseases (Age-Related Eye Disease Study [AREDS] and Carotenoids in Age-Related Maculopathy Italian Study [CARMIS]) [40,41], there are controversies about whether they have a protective effect on cellular oxidative stress model. In one study, ascorbic acid did not have a protective effect on Fenton-reaction-mediated oxidative stress model of ARPE-19 but it rather decreased the cell survival ratio at a low concentration (0.1–1 mM) compared to the group without ascorbic acid [42]. This was also the case for a *tert*-butyl hydroperoxide (t-BOOH)-induced oxidative stress model. In a study by Kagan et al., ascorbic acid (0.02–0.2 mM) also decreased the cell viability of ARPE-19 with oxidative stress induced by t-BOOH [43]. The effect of t-BOOH in porcine RPE also could not be diminished by ascorbic acid [44]. In our study, however, ascorbic acid increased the viability of the cells even at a low concentration where studies mentioned above suggest it decreases the viability and this was confirmed within two different oxidative stress models mediated by H_2_O_2_ and UVB. Although the central mechanism of t-BOOH to induce oxidative stress is by generating alkyl radicals [45], H_2_O_2_ is the central redox signaling molecule in general [30] forming hydroxy radicals [46], which can react intracellularly to generate various radicals including alkyl radical [47]. Considering H_2_O_2_ model reproduces more general situation of oxidative stress, and UVB model mediates H_2_O_2_ as the central signaling molecule [48], our result based on both models is more convincing. 

Ascorbic acid neutralized the effect of the oxidative stress inducer in both H_2_O_2_ and UVB model but astaxanthin only did so in UVB-induced stress model. Li et al. [38] investigated the effect of astaxanthin on ARPE-19 cells against oxidative stress with H_2_O_2_. They incubated ARPE-19 cells with 20 μM astaxanthin for different lengths of time (6, 12, and 24 h) and then exposed to 200 μM H_2_O_2_ for 24 h. The cell viability increase was time-dependent, and they suggested that 24 h was the optimal time for astaxanthin treatment. In the current study, we incubated ARPE-19 cells with ascorbic acid or astaxanthin for 6 h and then exposed to H_2_O_2_ with antioxidants for 24 h. Our findings that astaxanthin did not show significant effect on the cell viability with H_2_O_2_ exposure while it did show increased viability with UVB irradiation may be due to different lengths of time from those of Li et al. [38] for astaxanthin treatment. There is also another possibility that even if astaxanthin, an extremely lipophilic compound, was dissolved in DMSO, it may be possible that the efficiency was lower than the actual concentration in an aqueous environment such as cell culture medium.

The synergistic effect of ascorbic acid and astaxanthin was also evaluated in this study. The combination of ascorbic acid and astaxanthin showed better antioxidative effect compared to each drug alone. There are few reports that investigate the effective antioxidant action of ascorbic acid and astaxanthin in combination. In a study by Guerra et al. [49], the association of astaxanthin with ascorbic acid greatly improved neutrophil phagocytic capacity and decreased ROS with pro-inflammatory cytokines. They suggested that the astaxanthin/ascorbic acid system mimics the recycling system of vitamin E/vitamin C. Astaxanthin provides cell membranes with potent protection against free radicals or other oxidative attack. Moreover, previous studies confirm that astaxanthin has a large capacity to neutralize free radicals or other oxidant activity in the nonpolar zones of phospholipid aggregates, as well as along their polar boundary zones [50]. Ascorbic acid, in turn, promotes antioxidant effects mainly in a water-phase microenvironment. The exact mechanism other than reducing the intracellular ROS production is unknown through this study, but it is assumed that the two antioxidants exhibited synergistic effects through the mechanism identified in previous experimental studies.

An elevated level of ROS is always observed in the diabetic retina. Given that the administration of antioxidants in animal models preserves retinal capillaries from hyperglycemia-induced degeneration, ROS are considered a major causative factor involved in DR development [51,52]. Meanwhile, diabetes induces mitochondrial damage in the retina and its capillary cells and mitochondrial dysfunction is also considered to play a significant role in the development of DR [53,54]. The results of this study showed that antioxidants treatment resulted in significantly improved cell viability which is perhaps due to the improved mitochondrial function, improved cellular attachment performance, and increased growth rate of the cells. However, further investigation will be required to determine more precise mechanisms and effects of antioxidants on ARPE-19 cells.

Although the role of retinal endothelial cells comprising inner blood-retinal barriers (BRB) is important in DR development, the role of RPE cells comprising outer BRB is also crucial. The flow of nutrients materials, metabolites, ions, proteins, and water flux to and from the retina is regulated by these two BRBs and disruption of inner and outer BRB causes retinal hyperpermeability and development of diabetic macular edema, which is a leading cause of vision loss in DR [55]. Therefore, current study using ARPE-19 cells is thought be closely related to BRB breakdown and the pathogenesis of DR. Oxidative damage to cells is commonly modeled using treatment with H_2_O_2_ for a long time [56,57], however, very little is known about the role of UV irradiation on ARPE-19 cells and especially on the development of DR. A recent population-based study investigated the association between daily sunlight exposure duration and DR [58]. The authors suggested that the risk of DR was 2.66 times higher in the group with ≥ 5 h of daily sunlight exposure than in the group with less exposure after adjusting for risk factors such as duration of diabetes, serum hemoglobin A1c level, hypertension, and dyslipidemia. Although a lot of evidence is still lacking, the results of the current study can be used as evidence that the effects of oxidative stress induced by H_2_O_2_ or UVB irradiation on the development of DR as well as the antioxidants can reduce disruption of outer BRB, which is represented by the viability of ARPE-19 cells.

This study successfully established an oxidative stress model of RPE which can be used to test potential antioxidative compounds. Since it has been known that oxidative stress is closely linked to diabetic retinopathy, identifying antioxidants like ascorbic acid and astaxanthin used in this study can also be beneficial to patients with diabetic retinopathy.

## 5. Conclusions

In summary, the antioxidative effect of ascorbic acid and astaxanthin was evaluated in this study using two different oxidative stress models achieved by H_2_O_2_ and UVB. Despite controversies questioning the antioxidative property of ascorbic acid, it was shown in this study that ascorbic acid diminishes the oxidative damage within human RPE oxidative stress models of H_2_O_2_ and UVB which reflect general circumstance of oxidatively stressed environment. This study also showed the ROS scavenging capacity of astaxanthin in a UVB-induced stress model. Synergistic effect of ascorbic acid and astaxanthin was also shown resulting in increment in cell viability and reduction in intracellular ROS level.
